# Co-occurrence of Kikuchi-Fujimoto Disease, Systemic Lupus Erythematosus, and Macrophage Activation Syndrome Presenting With Acute Pancreatitis and Myositis in an Adolescent Male Patient

**DOI:** 10.7759/cureus.109513

**Published:** 2026-05-23

**Authors:** Amit R Rup, Kinnera Sailaja, Hema Latha Gundu, Keertthana Padmanaban, Swarnalata Das, Akruti Mishra, Nirmal K Mohakud

**Affiliations:** 1 Pediatrics, Kalinga Institute of Medical Sciences, Bhubaneswar, IND; 2 Pathology, Kalinga Institute of Medical Sciences, Bhubaneswar, IND; 3 Pediatric Medicine, Kalinga Institute of Medical Sciences, Bhubaneswar, IND

**Keywords:** adolescent, kikuchi-fujimoto disease, lymphadenopathy, macrophage activation syndrome, pancreatitis, systemic lupus erythematosus

## Abstract

Kikuchi-Fujimoto disease (KFD) is a rare, self-limiting necrotizing lymphadenitis that can mimic infection, malignancy, or autoimmune disease. Its coexistence with systemic lupus erythematosus (SLE) and macrophage activation syndrome (MAS) is uncommon and presents significant diagnostic challenges. We report a 15-year-old boy who presented with prolonged fever, generalized lymphadenopathy, cytopenias, oral ulcers, myositis, and acute pancreatitis. Laboratory evaluation revealed positive antinuclear and anti-Smith antibodies, hypocomplementemia, markedly elevated serum ferritin levels (27,650 ng/mL), hypertriglyceridemia, and hypofibrinogenemia. Lymph node biopsy demonstrated histiocytic necrotizing lymphadenitis consistent with KFD. The patient fulfilled the diagnostic criteria for SLE complicated by MAS and acute pancreatitis. Early initiation of high-dose corticosteroids, intravenous immunoglobulin, cyclophosphamide, cyclosporine, and hydroxychloroquine resulted in clinical and biochemical remission. This case highlights the importance of recognizing KFD-SLE overlap with MAS in adolescents presenting with persistent fever and cytopenias, as prompt immunosuppressive therapy can be life-saving.

## Introduction

Kikuchi-Fujimoto disease (KFD), first described in Japan in 1972, is a histiocytic necrotizing lymphadenitis of unknown etiology that typically presents with fever and cervical lymphadenopathy in young adults, particularly females of Asian descent [1]. Although generally benign and self-limiting, KFD poses a diagnostic challenge because it can mimic tuberculosis, lymphoma, and autoimmune disorders owing to its nonspecific clinical presentation [2].

Systemic lupus erythematosus (SLE) is a multisystem autoimmune disease characterized by heterogeneous clinical manifestations, including lymphadenopathy and cytopenias [3]. Increasing evidence suggests a clinicopathological association between KFD and SLE, with KFD preceding, coexisting with, or following the diagnosis of lupus in approximately 20%-30% of cases [4]. The underlying mechanism linking these conditions remains incompletely understood but is thought to involve immune dysregulation and shared genetic susceptibility.

Macrophage activation syndrome (MAS), a secondary form of hemophagocytic lymphohistiocytosis (HLH), is a life-threatening hyperinflammatory complication associated with autoimmune diseases, including SLE [5]. It is characterized by cytopenias, hyperferritinemia, hypofibrinogenemia, and multiorgan dysfunction, and carries substantial mortality if not recognized early [6]. The simultaneous occurrence of KFD, SLE, and MAS is exceptionally rare, particularly in the pediatric population, with only limited cases reported in the literature [7]. This rarity likely reflects the need for a convergence of specific genetic susceptibilities and environmental triggers, compounded by the diagnostic challenge of identifying localized necrotizing lymphadenitis in the setting of severe systemic hyperinflammation.

Recent advances in understanding the pathogenesis of these overlapping conditions have highlighted the role of cytokine dysregulation, particularly interferon-gamma (IFN-γ) and interleukin-18 (IL-18), in driving the hyperinflammatory state [8]. Defective clearance of apoptotic cells, a shared feature of both KFD and SLE, may result in persistent macrophage activation and excessive IL-18 production. Elevated IL-18 subsequently stimulates cytotoxic T cells to release large amounts of IFN-γ, which further activates tissue macrophages in a self-perpetuating inflammatory loop. This uncontrolled “cytokine storm” contributes to the severe cytopenias, hemophagocytosis, and multiorgan dysfunction characteristic of MAS [9,10]. The 2019 EULAR/ACR classification criteria for SLE and the 2016 classification criteria for MAS complicating rheumatic diseases have improved diagnostic accuracy and facilitated earlier therapeutic intervention [11,12]. In particular, the 2019 SLE criteria employ a weighted scoring system that better captures early or atypical disease presentations, while the 2016 MAS criteria emphasize dynamic changes in biomarkers such as extreme hyperferritinemia and rapidly declining blood counts, enabling earlier recognition of evolving hyperinflammation and timely initiation of life-saving immunosuppressive therapy.

We describe a rare case of concurrent KFD, SLE, and MAS in an adolescent male patient with multiorgan involvement, including acute pancreatitis and myositis, highlighting the diagnostic complexity, therapeutic challenges, and importance of early multidisciplinary management.

## Case presentation

A 15-year-old previously healthy boy presented with high-grade intermittent fever for 25 days, progressive periorbital edema, generalized weakness, painless oral ulcers, and neck swelling. The fever was associated with chills and rigors, although he remained active during afebrile intervals. He had previously received multiple courses of antibiotics from local practitioners for a suspected urinary tract infection without clinical improvement.

Ten days before admission, his family noted bilateral periorbital swelling that was more prominent in the morning, decreased urine output, and progressive neck swelling that initially appeared pea-sized and gradually enlarged to approximately lemon size. Five days prior to presentation, he developed generalized weakness with pain on movement, multiple painless oral ulcers over the soft palate, generalized colicky abdominal pain, and a papular rash over the upper chest.

There was no history of joint pain or swelling, headache, bleeding manifestations, night sweats, significant weight loss, cough, or breathlessness. No prior autoimmune illness or significant drug exposure was reported.

On admission, the patient was febrile (38.3°C) but hemodynamically stable, with a blood pressure of 110/64 mmHg, pulse rate of 86 beats/min, and respiratory rate of 18/min. General physical examination revealed pallor, bilateral periorbital edema, and subconjunctival hemorrhage in the right eye. Oral examination showed a 0.5 × 0.5 cm painless ulcer on the soft palate with overlying candidiasis. Multiple bilateral cervical lymph nodes (the largest measuring 2 × 1.5 cm) and left axillary lymphadenopathy were palpable; the nodes were firm and non-tender.

Musculoskeletal examination demonstrated proximal muscle weakness with muscle power graded 3/5 in all four limbs. Neck stiffness with a positive Kernig’s sign was noted. Dermatological examination revealed a papular rash over the upper chest. Hepatosplenomegaly was absent on initial examination. Anthropometric assessment revealed underweight status, with a body mass index (BMI) of 15.9 kg/m². Tanner staging was stage IV, appropriate for age.

Given the presentation of prolonged fever, generalized lymphadenopathy, cytopenias, and multisystem involvement, the differential diagnoses considered included hematological malignancy (lymphoma/leukemia), owing to generalized lymphadenopathy, cytopenias, and elevated lactate dehydrogenase (LDH); tuberculosis, which is endemic in our region and commonly presents with prolonged fever and lymphadenopathy; SLE, because of multisystem involvement including oral ulcers, cytopenias, and renal manifestations; HLH/MAS, in view of persistent cytopenias and hyperferritinemia; KFD, considering the possibility of necrotizing lymphadenitis associated with fever; and infectious etiologies (viral, bacterial, and tropical infections) causing prolonged febrile illness with lymphadenopathy.

Laboratory investigations revealed progressive pancytopenia with declining hemoglobin, leukocyte, and platelet counts, accompanied by low complement levels (C3 and C4) and disproportionately low inflammatory markers. In contrast, the urine protein-creatinine ratio, creatine phosphokinase (CPK), and LDH levels were markedly elevated. Initial liver function tests demonstrated mild transaminitis, with aspartate aminotransferase (AST) of 122 U/L and alanine aminotransferase (ALT) of 147 U/L (reference range: <40 U/L), consistent with systemic hyperinflammation, while serum electrolytes remained within normal limits. Immunological evaluation was positive for antinuclear antibody (ANA), anti-Smith, ribosomal P, nucleosome, and Jo-1 antibodies. The patient ultimately fulfilled 11 of the 17 SLICC classification criteria for SLE, confirming the diagnosis [13] (Table 1).

**Table 1:** Laboratory findings at presentation and during the course of hospitalization ANA: antinuclear antibody, C3/C4: complement components 3 and 4, Ig: immunoglobulin. Note: Values represent laboratory parameters obtained at different time points during the illness, corresponding to clinically indicated evaluations.

Parameter	Values during hospitalization	Reference range
Hemoglobin	8.2 g/dL (nadir)	12-16 g/dL
Total leukocyte count	3,420/µL	4,000-11,000/µL
Platelet count	90,000/µL	150,000-450,000/µL
Urine protein/creatinine ratio	1.83	<0.2
Serum creatinine	0.8 mg/dL	0.6-1.2 mg/dL
ANA titer	Positive (1:80)	Negative
Anti-Smith antibody	Positive (2+)	Negative
Ribosomal P antibody	Strongly positive (3+)	Negative
Complement C3/C4	Low	Normal
Ferritin	27,650 ng/mL	20-300 ng/mL
Triglycerides	288 mg/dL	<150 mg/dL
Fibrinogen	128.6 mg/dL	200-400 mg/dL
Creatine phosphokinase	4,098 U/L	30-200 U/L
Lactate dehydrogenase	2,614 U/L	140-280 U/L
Amylase	667 U/L	30-110 U/L
Lipase	8,954 U/L	0-160 U/L
C-reactive protein	0.4 mg/L	<5 mg/L
Procalcitonin	0.33 ng/mL	<0.5 ng/mL
IgA	220 mg/dL	70-400 mg/dL
IgG	2,262 mg/dL	700-1,600 mg/dL
IgM	167 mg/dL	40-230 mg/dL

Given the persistence of cytopenias despite a negative sepsis workup and a falling erythrocyte sedimentation rate (ESR) after one week of admission, MAS was suspected. Further evaluation revealed marked hyperferritinemia (27,650 ng/mL), hypertriglyceridemia (288 mg/dL), and hypofibrinogenemia (128.6 mg/dL), supporting the diagnosis of MAS complicating SLE. According to the HLH-2004 diagnostic criteria, the patient fulfilled five of the eight required criteria: fever, cytopenias involving at least two cell lineages, hypertriglyceridemia, hypofibrinogenemia, and hyperferritinemia [14].

Evaluation of his abdominal pain demonstrated markedly elevated serum amylase (667 U/L) and lipase (8,954 U/L) levels. Contrast-enhanced computed tomography (CT) of the pelvis and abdomen revealed ascites, hepatosplenomegaly, and a bulky pancreatic tail, findings consistent with acute pancreatitis (Figures 1, 2).

**Figure 1:**
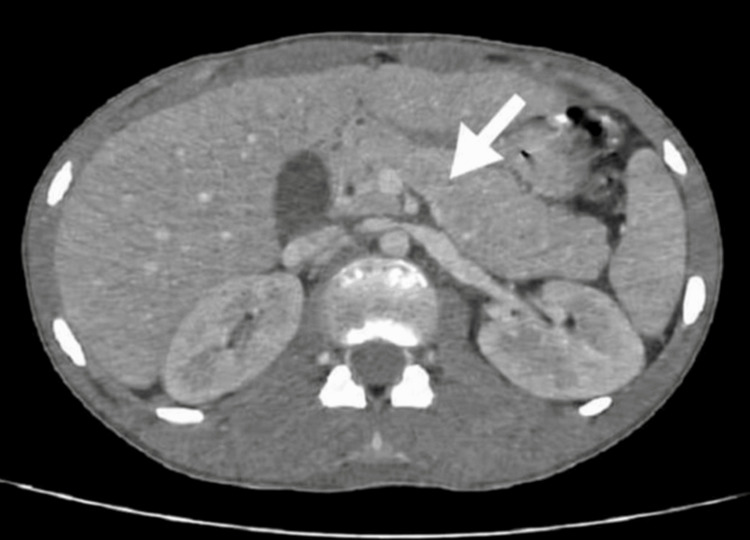
Contrast-enhanced CT of the abdomen demonstrating acute pancreatitis with associated hepatosplenomegaly Axial contrast-enhanced CT image of the abdomen showing features consistent with acute pancreatitis. There is a bulky appearance of the pancreatic tail (arrow), with surrounding inflammatory changes. Associated findings include ascites and enlargement of both the liver and spleen (hepatosplenomegaly), supporting the diagnosis of acute pancreatitis with systemic involvement.

**Figure 2:**
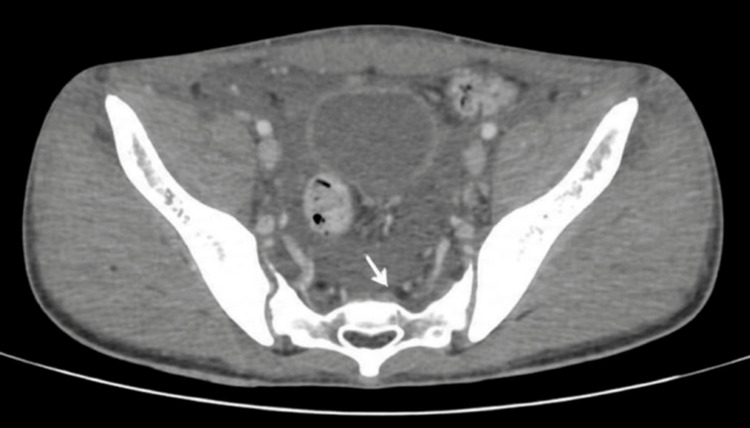
Contrast-enhanced CT of the pelvis demonstrating ascites associated with acute pancreatitis Axial contrast-enhanced CT of the pelvis from the same patient demonstrating free intraperitoneal fluid consistent with ascites (arrow). The fluid is seen layering within the dependent pelvic cavity, supporting the presence of ascites as part of the systemic manifestations of acute pancreatitis.

A systematic and stepwise diagnostic approach was undertaken to differentiate among infectious, malignant, and autoimmune etiologies in this patient presenting with prolonged fever, cytopenias, and multisystem involvement. Infectious causes, including tuberculosis and other bacterial or viral infections, were excluded through an extensive negative microbiological workup, including sterile blood and urine cultures, negative cartridge-based nucleic acid amplification testing (CBNAT) for *Mycobacterium tuberculosis*, and unremarkable viral serologies. Hematological malignancies such as lymphoma and leukemia were ruled out by bone marrow examination, which showed no evidence of malignant infiltration, along with negative immunophenotyping and flow cytometry studies. In view of the meningeal signs, cerebrospinal fluid (CSF) analysis was performed and was within normal physiological limits except for mildly elevated protein levels (58 mg/dL), thereby excluding acute infectious meningitis.

Excisional lymph node biopsy demonstrated classical histopathological features of KFD, including histiocytic aggregates with plasma cells and lymphocytes in the paracortical regions, abundant karyorrhectic debris, and absence of significant neutrophilic infiltration. Many histiocytes exhibited peripherally compressed crescent-shaped nuclei and contained phagocytosed cellular debris (Figure 3). Immunohistochemistry showed positivity for CD3, CD4, CD8, CD20, CD68, and myeloperoxidase (MPO), supporting the diagnosis of histiocytic necrotizing lymphadenitis consistent with KFD.

**Figure 3:**
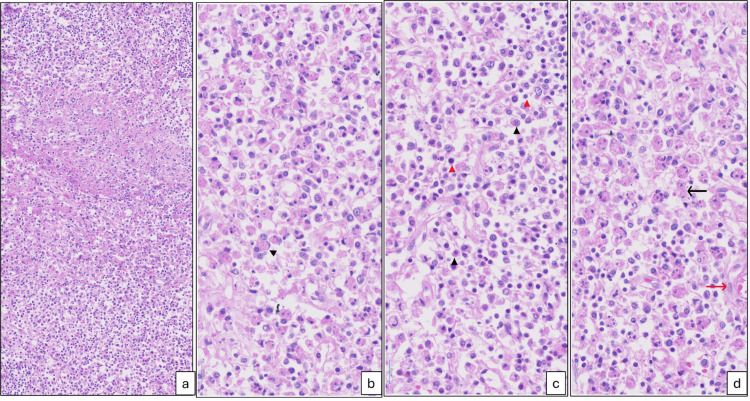
Histopathological findings of Kikuchi-Fujimoto disease on lymph node biopsy: histiocytic aggregates, karyorrhexis, and crescentic nuclei (H&E, 400X) (a) Low-power microscopic view showing distortion of the lymph node architecture with pale areas of histiocytic aggregates. (b) Aggregates of histiocytes, lymphocytes, and plasma cells. Many of the histiocytes show peripherally compressed crescentic nuclei (black arrowhead). (c) High-power magnification showing plasma cells (black arrowhead) and lymphocytes (red arrowhead), with absence of neutrophils. (d) High-power microscopic view showing histiocytes containing engulfed karyorrhectic debris (black arrow) and plump endothelial cells lining the blood vessels (red arrow).

Empirical antibiotic therapy with ceftriaxone, later escalated to meropenem and linezolid, was discontinued after infectious etiologies were excluded. The patient received pulse corticosteroid therapy with intravenous methylprednisolone for three days; however, owing to persistent inflammatory activity reflected by ongoing laboratory abnormalities, treatment was extended to a total of six days. Intravenous immunoglobulin (IVIG) was subsequently administered at a dose of 2 g/kg over three days [15].

Cyclosporine was initiated as adjunctive immunosuppressive therapy but was temporarily withheld because of worsening leukopenia and later resumed following hematological improvement. Intravenous cyclophosphamide was administered at a dose of 15 mg/kg with mesna according to the National Institutes of Health (NIH) protocol for severe lupus, with a plan for six monthly pulses; it was not continued as daily oral therapy. For long-term maintenance therapy, hydroxychloroquine was continued indefinitely at 5 mg/kg/day, while oral prednisolone at 1 mg/kg/day was maintained for four weeks, followed by a gradual taper over six months to the lowest effective dose.

Acute pancreatitis was managed conservatively with bowel rest, analgesia, and serial monitoring of pancreatic enzymes and liver function tests, all of which gradually normalized over a period of three weeks.

Ophthalmologic evaluation for blurred vision revealed subconjunctival ecchymosis and two cotton wool spots on fundoscopic examination, findings consistent with lupus retinopathy. Conservative ocular management was advised.

During a total hospital stay of 40 days, the patient demonstrated gradual but sustained clinical improvement. Fever subsided, cytopenias resolved, ferritin levels decreased from 27,650 ng/mL to 850 ng/mL, pancreatic enzyme levels normalized, and muscle strength returned to baseline (power 5/5 in all limbs). At discharge, he was clinically stable on maintenance immunosuppressive therapy with hydroxychloroquine and tapering oral prednisolone, with close rheumatology follow-up arranged.

At the three-month follow-up, the patient remained in clinical and biochemical remission without evidence of disease flare or relapse. The timeline of evolving multisystem involvement and the stepwise diagnostic and therapeutic approach in this rare overlap of KFD, SLE, and MAS is illustrated in Figure 4.

**Figure 4:**
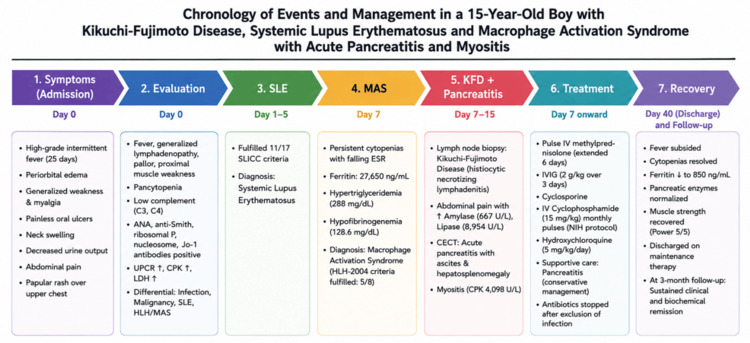
Chronological timeline of clinical events, diagnostic workup, and management from admission (day 0) to recovery SLE: systemic lupus erythematosus, MAS: macrophage activation syndrome, KFD: Kikuchi-Fujimoto disease, CPK: creatine phosphokinase, LDH: lactate dehydrogenase, UPCR: urine protein/creatinine ratio, ANA: antinuclear antibody, CECT: contrast-enhanced computed tomography, IVIG: intravenous immunoglobulin. Note: This figure was generated with assistance from ChatGPT (OpenAI, San Francisco, CA, USA; accessed May 2026) and subsequently reviewed and edited by the authors for clinical accuracy.

## Discussion

The novelty of this case lies in the simultaneous presentation of KFD, SLE, and MAS in an adolescent male patient, with additional uncommon manifestations including acute pancreatitis and myositis, initially mimicking malignancy. While KFD is classically described as a benign, self-limiting condition predominantly affecting young Asian women, this case demonstrates that its association with SLE can herald a severe systemic hyperinflammatory state requiring aggressive immunomodulatory therapy [16].

To our knowledge, this represents one of the few reported pediatric cases documenting the complete triad of KFD, SLE, and MAS with concurrent pancreatitis and myositis. The diagnostic complexity was compounded by the patient’s age and gender (adolescent male), as both KFD and SLE show female predominance, potentially delaying consideration of these diagnoses. The strong female predominance in these overlapping autoimmune and hyperinflammatory conditions is widely thought to be driven by a combination of estrogen-mediated immune responses, such as enhancement of autoreactive B-cell survival, and X-linked genetic susceptibility, making this severe presentation in a male particularly unusual [17,18]. The extreme hyperferritinemia (27,650 ng/mL) and multiorgan involvement reflect the severity of immune dysregulation when these conditions overlap, challenging the traditional view of KFD as uniformly benign [4,19].

Our therapeutic approach addressed three interconnected pathological processes: (1) the underlying SLE with pulse corticosteroids and cyclophosphamide, (2) the hyperinflammatory MAS with high-dose corticosteroids and IVIG, and (3) the associated KFD, which typically does not require specific therapy but, in this context, represented a manifestation of systemic autoimmunity. Cyclosporine was added based on its efficacy in MAS through inhibition of T-cell activation and cytokine production [20]. Hydroxychloroquine was continued as the cornerstone of long-term SLE management. The decision to use cyclophosphamide was guided by severe organ-threatening involvement, including lupus nephritis (urine protein/creatinine ratio 1.83) and CNS manifestations (neck stiffness, positive Kernig’s sign) [21].

Recognition of the KFD-SLE overlap has been facilitated by improved immunohistochemical techniques. In our case, CD68 and MPO positivity within necrotic areas helped distinguish KFD from lupus lymphadenitis, which may show similar histologic features but typically lacks the characteristic karyorrhectic debris and histiocytic proliferation [2]. The 2016 MAS classification criteria proved invaluable in early recognition, with our patient meeting criteria through hyperferritinemia, cytopenias, hypofibrinogenemia, and hypertriglyceridemia [10]. Recent studies have highlighted the utility of the ferritin-to-ESR ratio and serial ferritin monitoring in assessing MAS activity and treatment response [22].

Beyond conventional immunosuppression, biologic agents are emerging as therapeutic options for refractory MAS and severe SLE. Anakinra (IL-1 receptor antagonist) has shown promise in MAS complicating rheumatic diseases, although data in pediatric SLE-associated MAS remain limited [8]. Rituximab (anti-CD20) has been effective in refractory lupus and may have a role in KFD-SLE overlap, particularly when hematologic manifestations predominate. The recent approval of anifrolumab (anti-type I interferon receptor) for moderate-to-severe SLE represents a targeted approach that may benefit patients with interferon-driven disease, although its role in acute MAS requires further study [23].

Current research focuses on understanding the shared immunopathogenesis of KFD and SLE, with attention to type I interferon signatures, dysregulated apoptosis, and genetic predisposition. The shared pathophysiology among KFD, SLE, and MAS centers on defective clearance of apoptotic cells and profound cytokine dysregulation [10]. The massive apoptotic load and karyorrhectic debris characteristic of KFD can overwhelm the already impaired clearance mechanisms seen in SLE, exposing autoantigens and fueling systemic autoimmunity. This localized histiocytic activation in KFD may subsequently act as a catalyst for a systemic cytokine storm, driven primarily by interferon-gamma and interleukin-18, precipitating MAS [9].

Despite this shared immunopathogenic basis, the simultaneous occurrence of all three conditions remains exceptionally rare. We postulate that this rarity is multifactorial. First, it requires an unusual demographic and genetic alignment, particularly in an adolescent male, as both KFD and SLE are heavily female-predominant. Progression to MAS likely requires a concurrent underlying defect in cytolytic pathways combined with a specific environmental trigger. Second, diagnostic overshadowing may play a significant role; the severe cytopenias and coagulopathy defining MAS often preclude invasive procedures such as excisional lymph node biopsy, making concurrent KFD likely underreported in severe lupus flares.

The 2019 EULAR/ACR SLE classification criteria emphasize a weighted scoring system incorporating autoantibodies and clinical domains, thereby improving diagnostic sensitivity in pediatric populations [3]. For MAS, the 2016 classification criteria developed for systemic juvenile idiopathic arthritis are increasingly applied to SLE-associated MAS, although condition-specific criteria are still needed [14].

The importance of early tissue diagnosis cannot be overemphasized. In our patient, lymph node biopsy was critical in excluding lymphoma and tuberculosis while confirming KFD. Bone marrow examination ruled out primary HLH and malignancy. This systematic approach, guided by multidisciplinary collaboration among rheumatology, hematology, infectious diseases, and pathology teams, enabled timely diagnosis before irreversible organ damage occurred.

Recognition of MAS on day 7 of hospitalization, prompted by falling ESR with persistent cytopenias and extreme hyperferritinemia, allowed immediate intensification of immunosuppression. Serial monitoring of ferritin levels, cytopenias, and pancreatic enzymes guided treatment adjustments and confirmed response. By the time of discharge, the patient had achieved complete clinical and biochemical remission, avoiding the high mortality associated with untreated MAS.

This case illustrates that necrotizing lymphadenitis, when occurring in association with SLE, may present with extensive systemic involvement rather than the limited and self-resolving course often described. The presence of generalized lymphadenopathy, evolving cytopenias, and marked hyperferritinemia required careful exclusion of lymphoma, tuberculosis, primary hemophagocytic syndromes, and infection before a definitive diagnosis could be reached. Furthermore, the uncommon manifestations of acute pancreatitis and myositis required differentiation from drug-induced etiologies and primary viral infections, which was achieved through serial enzyme monitoring and correlation with systemic disease activity. Early recognition of MAS in this setting was crucial for the timely initiation of immunomodulatory therapy.

## Conclusions

KFD associated with SLE and MAS is rare but potentially life-threatening, particularly when complicated by pancreatitis and myositis, as seen in this adolescent. Overlapping clinical features with infection and malignancy often delay diagnosis, emphasizing the need for early histopathological confirmation and systematic evaluation. The extreme hyperinflammatory state in such overlap syndromes requires aggressive, multi-targeted immunosuppression guided by serial biomarker monitoring. A multidisciplinary approach, awareness of this overlap syndrome, and adherence to current classification criteria can facilitate timely recognition, reduce morbidity, and improve outcomes in this challenging patient population.

## References

[1] Kikuchi M (1972). Lymphadenitis showing reticulum cell hyperplasia with nuclear debris and phagocytes. Acta Hematol Jpn.

[2] Bosch X, Guilabert A, Miquel R, Campo E (2004). Enigmatic Kikuchi-Fujimoto disease: a comprehensive review. Am J Clin Pathol.

[3] Aringer M, Costenbader K, Daikh D (2019). 2019 European League Against Rheumatism/American College of Rheumatology classification criteria for systemic lupus erythematosus. Ann Rheum Dis.

[4] Baenas DF, Diehl FA, Haye Salinas MJ, Riva V, Diller A, Lemos PA (2016). Kikuchi-Fujimoto disease and systemic lupus erythematosus. Int Med Case Rep J.

[5] Ravelli A, Minoia F, Davì S (2016). 2016 classification criteria for macrophage activation syndrome complicating systemic juvenile idiopathic arthritis: a European League Against Rheumatism/American College of Rheumatology/Paediatric Rheumatology International Trials Organisation Collaborative Initiative. Ann Rheum Dis.

[6] Gavand PE, Serio I, Arnaud L (2017). Clinical spectrum and therapeutic management of systemic lupus erythematosus-associated macrophage activation syndrome: a study of 103 episodes in 89 adult patients. Autoimmun Rev.

[7] Liu C, Jin Y, Huang H (2022). Kikuchi-Fujimoto disease as the initial manifestation of systemic lupus erythematosus complicated with macrophage activation syndrome: two case reports and a review of literature. BMC Pediatr.

[8] Grom AA, Horne A, De Benedetti F (2016). Macrophage activation syndrome in the era of biologic therapy. Nat Rev Rheumatol.

[9] Crayne CB, Albeituni S, Nichols KE, Cron RQ (2019). The immunology of macrophage activation syndrome. Front Immunol.

[10] Andersson U (2021). Hyperinflammation: on the pathogenesis and treatment of macrophage activation syndrome. Acta Paediatr.

[11] Carter SJ, Tattersall RS, Ramanan AV (2019). Macrophage activation syndrome in adults: recent advances in pathophysiology, diagnosis and treatment. Rheumatology (Oxford).

[12] Uda K, Okubo Y, Matsushima T, Sadahira C, Kono T, Hataya H (2019). Multifocal infantile hemangioma. J Pediatr.

[13] Petri M, Orbai AM, Alarcón GS (2012). Derivation and validation of the Systemic Lupus International Collaborating Clinics classification criteria for systemic lupus erythematosus. Arthritis Rheum.

[14] Davì S, Minoia F, Pistorio A (2014). Performance of current guidelines for diagnosis of macrophage activation syndrome complicating systemic juvenile idiopathic arthritis. Arthritis Rheumatol.

[15] Borgia RE, Gerstein M, Levy DM, Silverman ED, Hiraki LT (2018). Features, treatment, and outcomes of macrophage activation syndrome in childhood-onset systemic lupus erythematosus. Arthritis Rheumatol.

[16] Rani R, Sharma N, Sharma V, Mahajan VK (2023). Kikuchi-Fujimoto disease: a comprehensive review. World J Clin Cases.

[17] Kim JW, Kim HA, Suh CH, Jung JY (2022). Sex hormones affect the pathogenesis and clinical characteristics of systemic lupus erythematosus. Front Med (Lausanne).

[18] Strickland FM, Hewagama A, Lu Q (2012). Environmental exposure, estrogen and two X chromosomes are required for disease development in an epigenetic model of lupus. J Autoimmun.

[19] Biswal S, Dash AK, Kumar M, Mohakud NK (2019). Kikuchi-Fujimoto disease in an adolescent girl with discoid skin lesion and high serum ferritin - a rare case report from Odisha. Indian J Child Health.

[20] Cruz-Pérez F, Vilá S, Ríos G, Vilá LM (2018). Efficacy of cyclosporine in the induction and maintenance of remission in a systemic lupus erythematosus patient presenting with macrophage-activating syndrome. Case Rep Rheumatol.

[21] Barile-Fabris L, Ariza-Andraca R, Olguin-Ortega L (2005). Controlled clinical trial of IV cyclophosphamide versus IV methylprednisolone in severe neurological manifestations in systemic lupus erythematosus. Ann Rheum Dis.

[22] Høeg PE, Glerup M, Mahler B, Høst C, Herlin T (2022). Evaluation of macrophage activation syndrome in patients with systemic juvenile idiopathic arthritis: a single center experience. Int J Rheumatol.

[23] Morand EF, Furie R, Tanaka Y (2020). Trial of anifrolumab in active systemic lupus erythematosus. N Engl J Med.

